# Toxicological and biochemical characterizations of AChE in phosalone-susceptible and resistant populations of the common pistachio psyllid, *Agonoscena pistaciae*

**DOI:** 10.1093/jis/14.1.18

**Published:** 2014-01-01

**Authors:** Ali Alizadeh, Khalil Talebi-Jahromi, Vahid Hosseininaveh, Mohammad Ghadamyari

**Affiliations:** 1 Department of Crop Protection, Faculty of Agriculture,Vali-e-Asr University of Rafsanjan, Iran; 2 Department of Plant protection, College of Agriculture and Natural Resources, University of Tehran, Karaj, Iran; 3 Department of Plant Protection, Faculty of Agriculture, University of Guilan, Iran

**Keywords:** AChE insensitivity, enzyme inhibition, kinetic parameters, organophosphate resistance

## Abstract

The toxicological and biochemical characteristics of acetylcholinesterases (AChE) in nine populations of the common pistachio psyllid, *Agonoscena pistaciae* Burckhardt and Lauterer (Hemiptera: Psyllidae), were investigated in Kerman Province, Iran. Nine *A. pistaciae* populations were collected from pistachio orchards, *Pistacia vera* L. (Sapindales: Anacardiaceae), located in Rafsanjan, Anar, Bam, Kerman, Shahrbabak, Herat, Sirjan, Pariz, and Paghaleh regions of Kerman province. The previous bioassay results showed these populations were susceptible or resistant to phosalone, and the Rafsanjan population was most resistant, with a resistance ratio of 11.3. The specific activity of AChE in the Rafsanjan population was significantly higher than in the susceptible population (Bam). The affinity ( *KM* ) and hydrolyzing efficiency ( *Vmax* ) of AChE on acetylthiocholine iodide, butyrylthiocholine iodide, and propionylthiocholine odide as artificial substrates were clearly lower in the Bam population than that in the Rafsanjan population. These results indicated that the AChE of the Rafsanjan population had lower affinity to these substrates than that of the susceptible population. The higher *Vmax* value in the Rafsanjan population compared to the susceptible population suggests a possible over expression of AChE in the Rafsanjan population. The *in vitro* inhibitory effect of several organophosphates and carbamates on AChE of the Rafsanjan and Bam populations was determined. Based on I50, the results showed that the ratios of AChE insensitivity of the resistant to susceptible populations were 23 and 21.7-fold to monocrotophos and phosphamidon, respectively. Whereas, the insensitivity ratios for Rafsanjan population were 0.86, 0.8, 0.78, 0.46, and 0.43 for carbaryl, eserine, propoxur, m-tolyl methyl carbamate, and carbofuran, respectively, suggesting negatively correlated sensitivity to organophosphate-insensitive AChE. Therefore, AChE from the Rafsanjan population showed negatively correlated sensitivity, being insensitive to phosphamidon and monocrotophos and sensitive to *N* -methyl carbamates.

## Introduction

The common pistachio psyllid, *Agonoscena pistaciae* Burckhardt and Lauterer (Hemiptera: Psyllidae), is a major pest of pistachio, *Pistacia vera* L. (Sapindales: Anacardiaceae), and is distributed in all pistachio producing areas of Iran ( Burckardt and Lauterer 1989 ; Lauterer 1998 ; Samih et al. 2005 ). The *A. pistaciae* populations are increasing in many countries, including Iran, Turkey, Iraq, Arme-nia, and Turkmenia, as well as in Mediterranean regions such as Syria and Greece ( Burckardt and Lauterer 1989 ; Burckhardt and Lauterer 1993; Mart et al.1995 ; Anagnou–Veroniki et al. 2008). Both adults and nymphs suck leaf sap and produce large amounts of white powder consisting of dried honeydew. Direct feeding of the pest causes reduced plant growth, defoliation, stunting, falling of fruit buds, and poor yield ( Lababidi 2002 ). Controlling *A. pistaciae* requires several insecticide treatments every year due to its short life span and high reproductive potential. Phosalone and amitraz are the insecticides most widely used to control *A. pistaciae* , although organophosphates, insect growth regulators, neonicotinoids, and pyrethroids have always been used in commercial pistachio orchards ( Samih et al. 2005 ).

Among the organophosphate insecticides, phosalone has been wieldy used against *A. pistaciae* , and its use is extended to the control of other pistachio pests, especially the pistachio leafhopper, *Idiocerus stali* Fieb. The resistance of *A. pistaciae* to phosalone has been pointed out by Telebi et al. (2001) and Alizadeh et al. (2011) . The synergists (TPP, PBO, and DEM) effect and activity of detoxifying enzymes (esterases, mixed function oxidases, and glutathione S-transferases) have demonstrated that the resistance to phosalone is mainly caused by esterase detoxification ( Alizadeh et al. 2011 ).

Acetylcholinesterase (AChE; EC 3.1.1.7), which hydrolyzes acetylcholine at the cholin-ergic synapse to terminating neurotransmission, is the major target site of both organophosphate and carbamate insecticides, which inhibit the enzyme activity by covalently phosphorylating or carbamylating the serine residue within the active site gap ( Corbett 1974 ; Fournier 2005 ). However, modification of AChE to an insensitive form can be related to the increased AChE activity and has been demonstrated as the most important mechanism providing resistance to the organophosphates and/or carbamates in some pests ( Fournier et al. 1992 , 1993; Zhu and Gao 1999 ; Kozaki et al. 2001 ). The kinetic microplate assay has been confirmed as a fast and accurate way of determining AChE-based resistant genotypes and is important both in the early detection of resistance and in the evaluation of other potential insecticides once this form of resistance has arisen ( Zhu and Gao 1999 ). The aims of the present research were to compare toxicological and biochemical characterizations of AChE among nine field populations of *A. pistaciae* from Kerman Province, Iran.

## Materials and Methods

Nine *A. pistaciae* populations were collected from pistachio orchards located in Rafsanjan, Anar, Bam, Kerman, Shahrbabak, Herat, Sirjan, Pariz, and Paghaleh regions of Kerman Province, Iran, in the summer of 2009. The populations were routinely reared in plastic boxes (50 ×60 ×80 cm) under greenhouse conditions at 28 ± 2ºC, 45 ± 5% RH, and a 16:8 L: D photoperiod on young, untreated pistachio plants, *P. vera* ( *var* . Badami Zaran-di). The Bam population was used as a reference susceptible population. The Rafsanjan population showed the highest resistance level (11.3) based on bioassay ( Alizadeh et al. 2011 ).

### Chemicals

S-butyrylthiocholione iodide (BTC), 5-5´-dithiobis-(2-nitrobenzoic acid) (DTNB), propionylthiocholine iodide (PTC), aldicarb (98% purity), carbofuran (99.5% purity), carbaryl (99.5% purity), and propoxure (99.6%) were purchased from Wako Pure Chemical Industries ( www.wako-chem.co.jp ). Phosphamidon and monocrotophos with 99.9% purity were purchased from Accustandard ( www.accustandard.com ). Acetylcholine iodide (ATC), eserine, tris (hydroxymethyl) aminomethane, Triton X-100, and bovine serum albumin were obtained from Sigma-Aldrich ( www.sigmaaldrich.com ).

### Enzyme preparation

Two hundred adults of *A. pistaciae* from each population were homogenized in 300 µL of icecold sodium phosphate buffer (10 mM, pH 7, containing 0.1% Triton X-100). The homogenates were centrifuged at 15,000 ×g and 4ºC for 10 min. The resulting supernatants were used as the enzyme source in all enzyme assays.

### Assay of AChE activities

AChE hydrolytic activities were measured by using ATC as the substrate, as described by Ellman et al. (1961) with some modifications ( Mamiya et al. 1961 ). The total reaction volume per well of a 96-well microtitre plate was 240 µL, consisting of 40 µL supernatant, 40 µL of ATC (1.5 mM) and DTNB (0.3mM), and 20 µL in 140 µL 0.1 M phosphate buffer (pH 7.0). The enzyme was preincubated at room temperature for 10 min. The absorbance was measured continuously every 5 min for 30 min at 405 nm and 37°C using a microplate reader (ELX808, BioTek, www.biotek.com ). The enzyme activities were calculated as described by Yin et al. (2001) .

### Determination of kinetic parameters of AChE

The apparent Michaelis–Menten constant ( *KM* ) and maximal velocity ( *Vmax* ) of total AChE from nine populations of *A. pistaciae* were determined according to Moores et al. (1996) with minor modifications. The activity was monitored continuously for at least 30 min with six different concentrations (0.1, 0.25, 2.5, 5, 10, and 25 µM) of ATC, BTC, and PTC. The detection method was the same as that used for the above activity assays. *KM* and *Vmax* values were calculated using the SigmaPlot 10.0 software ( www.sigmaplot.com ) for Windows, using the Michaelis–Menten equation according to the method of Wilkinson (1961) .

### Protein contents assay

The protein content of the enzyme samples was determined following the method of Lowry et al. (1951) using bovine serum albumin as the standard. The measurement was performed at a wavelength of 595 nm.

### *In vitro* inhibition of AChE

To discover whether the evaluated levels of AChE-specific activity present in the Rafsanjan population play a role in resistance to phosalone, eight chemicals from two different classes (organophosphates: phosphamidon, monocrotophos, and paraoxon; carbamates: propoxure, carbaryl, aldicarb, carbofuran, and eserine) were chosen to assess *in vitro* inhibition of AChE. The enzyme was preincubated with the inhibitor at room temperature for 10 min. After preincubation, the ATC, BTC, and PTC substrates were added to the mixture (containing 0.1 M phosphate buffer (pH 7.0) and DTNB). Stock solutions of the inhibitors were prepared in acetone and diluted with phosphate buffer (0.1 M, pH 7.0), thus the maximum acetone concentration was below 1% in the test solutions. The remaining activity was determined 20 min after the preparation of the reaction mixture. Optical density was measured at 405 nm with a microplate reader (ELX808, BioTek). The insecticide concentration inhibiting 50% of AChE activity (I50) was estimated using a re-gression-based approach, three parameters sigmoid, or logistical model. Each experiment was carried-out in triplicate. Calculations were performed using SigmaPlot10 and Microsoft Excel ( www.microsoft.com ).

### Analysis

Data were analyzed by employing ANOVA, and means were compared by Duncan’s multiple range test ( *P* < 0.05) using SPSS (IBM, www.ibm.com ).

## Results

### Assay of AChE activities

The activities of AChE in nine populations were measured with ATC as a substrate, and the results are presented in Table 1 . The results indicated that there were significant differences in AChE activities among the populations. The activity of AChE in the Rafsanjan population (the resistant population) was 1.95 fold higher than that of the Bam population (the susceptible population), and after Rafsanjan population, the AChE activity in Kerman and Sirjan populations showed the highest ratio. The rank order of the AChE activity, from the highest to the lowest, was Shahrbabak > Pariz > Anar > Herat > Paghaleh.

**Table 1.**
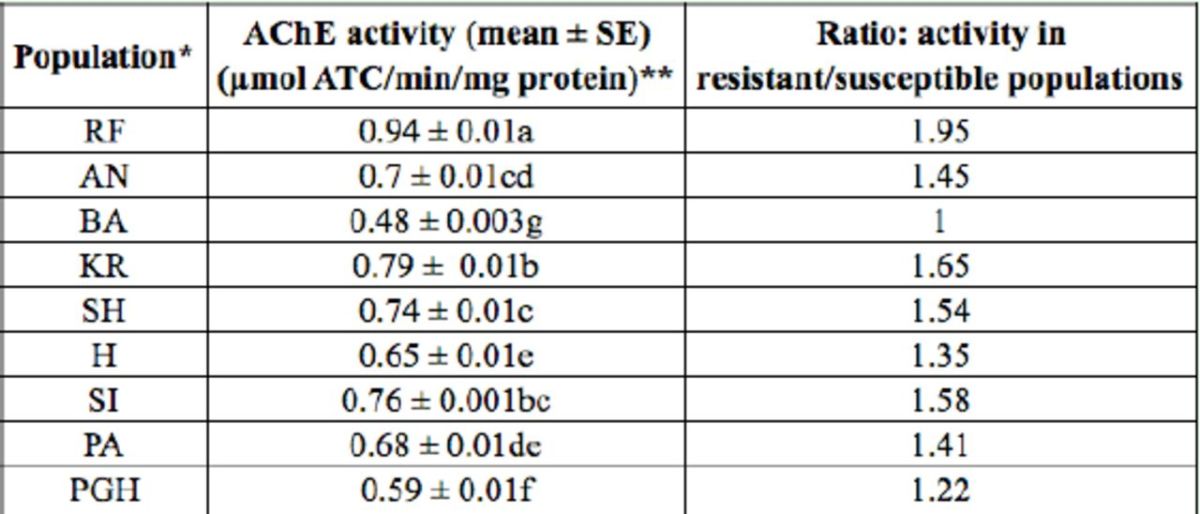
Comparison of AChE activities in nine populations of *Agonoscena pistaciae* .

*RF: Rafsanjan; AN: Anar; BA: Bam; KR: Kerman; SH: Shahrbabak; HE: Heart; SI: Sirjan; PA: Pariz; PGH: Paghaleh

**Means followed by different letters are significantly different at *P* < 0.05. Data were analyzed by employing ANOVA, and means were compared by Duncan’s multiple range test ( *P* < 0.05) using SPSS. ATC: acetylcholine iodide

### Kinetics analysis of AChE

Kinetic parameters of AChE from the nine populations of *A. pistaciae* to three substrates are shown in Table 2 . The rank order from the highest hydrolyzing efficiency to the lowest as expressed by the *Vmax* value was ATC > PTC > BTC for each population. The *KM* values of AChE in the resistance population (Rafsanjan) were 2.5, 2.51, and 2.98 fold higher than the susceptible population (Bam) with ATC, BTC, and PTC as substrates, respectively. The AChE of the Bam population (i.e., lower *K* M value, 0.24 µM) had significantly greater affinity to the substrate ATC than that of the Rafsanjan population ( *KM* =3.4 µM) ( *P* < 0.05). The *Vmax* value for the Rafsanjan population was 1.5, 1.8, and 1.6 fold higher for ATC, BTC, and PTC, respectively, than that for the Bam population. The catalytic activity of Bam AChE to ATC, BTC, and PTC, as expressed by the *Vmax* / *KM* values, indicated that they were significantly greater than the values for the Rafsanjan population ( Table 2 ).

**Table 2.**
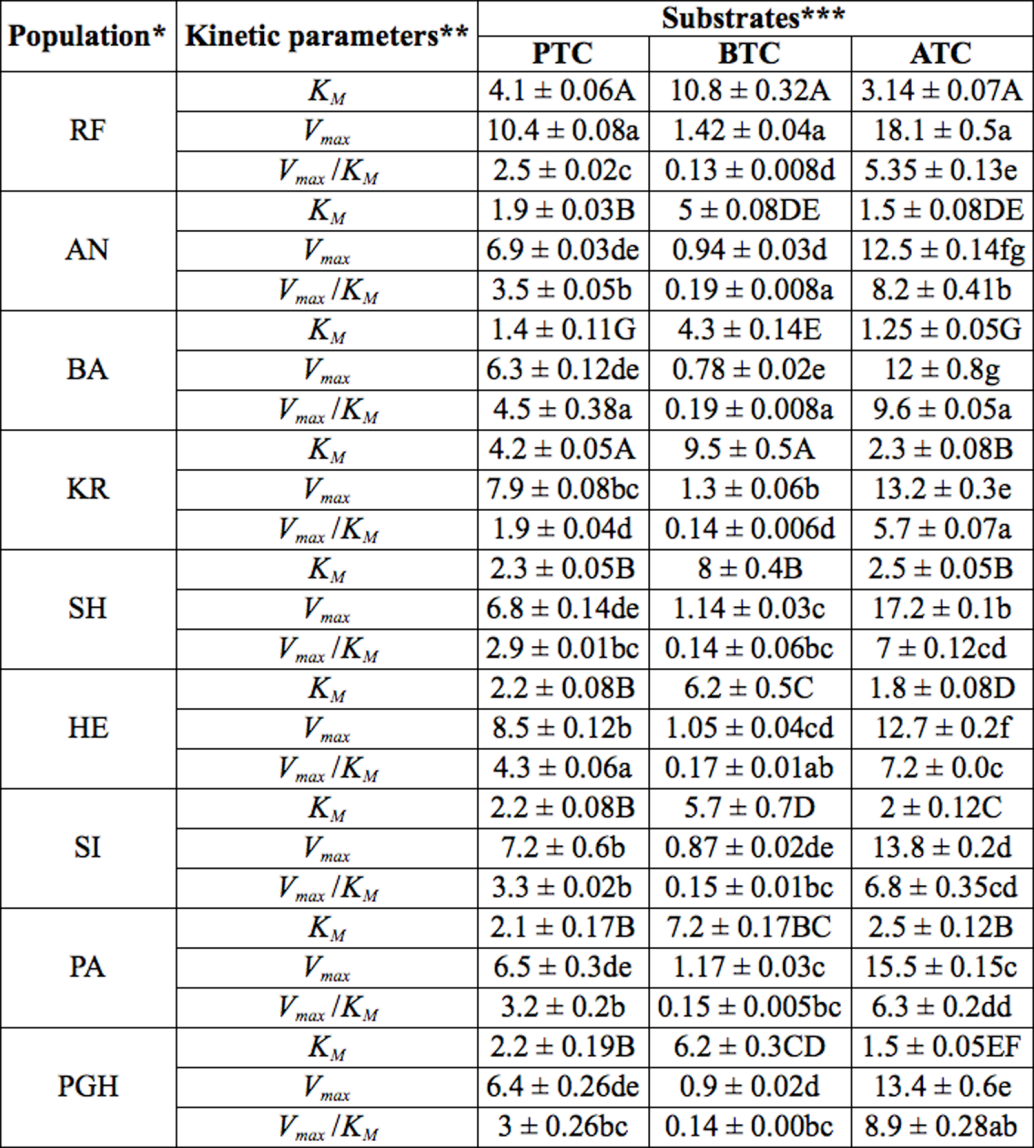
Substrate specificities and kinetic parameters of AChE from susceptible and field populations of *Agonoscena pistaciae* .

Each value represents mean (± SE) of three replications, and the means within the same column followed by different letters are significantly different at *P* < 0.05.

*RF: Rafsanjan; AN: Anar; BA: Bam; KR: Kerman; SH: Shahrbabak; HE: Heart; SI: Sirjan; PA: Pariz; PGH: Paghaleh

**The apparent Michaelis–Menten constant ( *KM* ) and maximal velocity ( *Vmax* ) are expressed as μ M and μ M/min/mg protein, respectively

***ATC: acetylcholine iodide; BTC: S-butyrylthiocholione iodide; PTC: propionylthiocholine iodide

### *In vitro* inhibition of AChE

The effects of organophosphates and carbamates on AChE activity from mass homogenates of resistant and susceptible populations were determined, and the results are shown in Table 3 . Compared with I50 values for the susceptible population (Bam), the resistant population (Rafsanjan) indicated 23, 21.7, and 1.1 fold resistance to monocrotophos, phosphamidon, and aldicarb, respectively. In contrast, the insensitivity factor of AChE from the Rafsanjan population was 0.86, 0.8, 0.78, 0.46, and 0.43 for carbaryl, eserine, propoxur, m-tolyl methyl carbamate (MTMC), and carbofuran, respectively. These results showed that resistance to phosalone in Rafsanjan population causes simultaneous sensitivity to inhibitors.

**Table 3.**
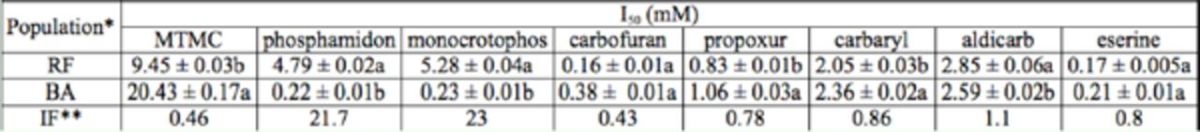
I50 values of *in vitro* inhibition of AChE activity in the most phosalone-resistant and susceptible populations of *Agonoscena pistaciae* .

*RF: Rafsanjan; BA: Bam

**Insensitivity factor, determined by dividing the I50s of resistant populations by the I50s of susceptible populations.

## Discussion

The relationship between AChE alteration and insect resistance (especially to carbamates and organophosphates) has been investigated in numerous studies ( Zhu and Gao 1999 ; Stumpf et al. 2001 ; Li and Han 2002 ; Yu 2006 ). A preliminary study on metabolic resistance mechanisms to phosalone in field populations of *A. pistaciae* has been performed by Alizadeh et al. (2011). Their synergism and biochemical experiments demonstrated the involvement of esterase in the phosalone resistance of *A. pistaciae* . In our current study, toxicological and biochemical characterizations of AChE in the common pistachio psyllid revealed that AChE was involved in *A. pistaciae* resistance. The results showed significant differences in the total AChE activity between the susceptible and the most phosalone-resistant population. However, no significant differences in AChE activity between resistant and susceptible populations were found in several cases ( Zhu and Brindley 1990 ; Tsagkarakou 2002 ). Many researchers have reported a relationship between insecticide resistance and a decrease in AChE activity ( Tang et al.1990 ; Vontas et al. 2001 ). In contrast, an increase in the activity of AChE was reported for the resistant strains in some cases ( Gao and Zhu 2000 ; Chai et al. 2007 ). In the present study, the biochemical characterization studies of AChE from the susceptible and resistant populations indicated higher specific activities of AChE in the Rafsanjan population than in the Bam population. Likewise, northern blot analysis suggested high activity resulting from over expression of AChE in *Schizaphis graminum* ( Zhu and Gao 1999 ). However, in our study some correlation between the development of resistance and the quantity or quality of AChE in *A. pistaciae* was observed.

The affinity ( *K _M_* ) and hydrolyzing efficiency ( *V _max_* ) are two important kinetic parameters of any enzyme. High *K _M_* values of AChE from the resistant population (Rafsanjan) implied that the AChE had lower affinity to all substrates (ATC, BTC, and PTC).The results of our study are similar to those observed from studies on *Liposcelis bostrychophila* ( Chai et al. 2007 ), *Leptinotarsa decemlineata* ( Zhu and Clark 1994 ), *Schizaphis graminum* ( Gao and Zhu 2001 ), and *Bactrocera dorsalis* ( Hsu et al. 2008) . The *V _max_* of AChE in the resistant populations may decrease ( Raymond et al. 1986 ; Hsu et al. 2008 ), increase ( Ren et al. 2002 ; Yu 2006 ), or remain without significant change ( Stumpf et al. 2001 ). In comparison, the *V _max_* value of the AChE from the Bam population was significantly lower than that of the Rafsanjan population. The higher *V _max_* values of AChE in the Rafsanjan population with ATC, BTC, and PTC substrates suggest possible overexpression of this enzyme in the resistant population. A similar phenomenon has also been reported for many other resistant insects ( Fournier et al. 1992 , 1993; Gao and Zhu 2002 ). However, kinetic characteristics of AChE between the Bam and Rafsanjan populations of *A. pistaciae* were different. In the Rafsanjan population, AChE activity, *K _M_* , and *V _max_* for the ATC, BTC, and PTC substrates were nearly two fold higher compared to those from the BA population. This may be due to the alteration of AChE catalytic site ( Berrada et al. 1994 ) in the *A. pistaciae* resistant population. On the other hand, the difference in kinetic parameters between the Bam and Rafsanjan populations was probably due to the combined results of the insensitive AChE, higher specific activity, and other factors.

Alteration of AChE has been observed with a reduced sensitivity to inhibition by organophosphate and carbamate insecticides in numerous insects and mites ( Stumpf et al. 2001 ; Gao and Zhu 2002 ; Hsu et al. 2008 ;). *In vitro* inhibition assays (I _50_ ) showed that AChE from the resistant population had higher sensitivity to carbofuran, MTMC, propoxur, eserine and carbaryl insecticides than AChE from the susceptible population. Therefore, negatively correlated sensitivity was found in the resistant population towards carbofuran and MTMC. So far, the studies on the resistance level and resistance spectrum of *A. pistaciae* to different insecticides were quite limited. However, negatively correlated sensitivity has also been reported in altered AChE of other insects ( Zhu and Clark 1995 ; Villatte et al. 1999 ; Ghadamyari et al. 2008 ).

Anti-resistant insecticides, which are more effective against resistant than susceptible genotypes, have been proposed as an important strategy for insecticide resistance management. The results of our study showed that carbofuran, MTMC, and propoxur were more active against modified AChE of *A. pistaciae* compared with sensitive AChE *.* Therefore, we report a modified AChE in Rafsanjan population of *A. pistaciae* showed negatively-correlated sensitivity, being insensitive to phosphamidon and monocrotophos and sensitive to *N* -methyl carbamates. Similar results were obtained by Ghadamyari et al. (2008) on pirimicarb resistant population of *Myzus persicae* . Also, Hama et al. (1980) found that AChE of the green rice leafhopper, *Nephotettix cincticeps* , has become insensitive to widely used *N* -methyl carbamates but hy-persensitive to *N* -propyl carbamate compounds. *In vivo* results of Villatte et al. (1999) showed that a pirimicarb resistant strain of *Aphis gossypii* was 2.5 fold more susceptible to bendiocarb than a sensitive strain. Therefore, pirimicarb resistant strain of *A. gossypii* showed negatively correlated cross-resistance to *N* -dimethyl and *N* -methyl carbamete ( Villatte et al. 1999 ).

Based on I50, *N* -methyl carbamete is expected to show high efficiency against the Rafsanjan population. However the effect of other mechanisms (i.e,. esterases, MFO, and GST) should not be ignored ( Alizadeh et al. 2011 ). In most cases, molecular investigations suggested that altered AChEs arise from point mutations in the gene encoding this enzyme, resulting in amino acid substitutions in the AChE catalytic center or near the active site of enzyme (Nabeshima et al. 2003; Cassanelli et al. 2006 ; Hsu et al. 2006 ; Kono et al. 2006 ). This gives the impression that these types of biochemical test will be difficult to develop for individual psylla because of their small size. In conclusion, AChE from the Rafsanjan population had lower affinity to artificial substrates (i.e., ATC, BTC and PTC) and reduced sensitivity to inhibition by phosphamidon and monocrotophos compared with susceptible populations, suggesting that the Rafsanjan population possessed qualitatively modified AChE. Therefore, developments in the molecular characterization of the target AChE gene are needed to advance tests based on molecular biology techniques, especially PCR.

## Abbreviations

ATC: acetylcholine iodide
BTC: S-butyrylthiocholione iodide
DTNB: 5-5´-dithiobis-(2-nitrobenzoic acid)
MTMC: m-tolyl methyl carbamate
PTC: propionylthiocholine iodide
